# Mitigating aflatoxin exposure to improve child growth in Eastern Kenya: study protocol for a randomized controlled trial

**DOI:** 10.1186/s13063-015-1064-8

**Published:** 2015-12-03

**Authors:** Vivian Hoffmann, Kelly Jones, Jef Leroy

**Affiliations:** International Food Policy Research Institute (IFPRI), 2033 K St. NW, Washington, DC USA

**Keywords:** Aflatoxin, Length-for-age Z-score (LAZ), Child growth, Stunting, Cluster-randomized trial

## Abstract

**Background:**

While the few studies that have looked at the association between stunting and aflatoxin exposure have found surprisingly large effects, the results remain inconclusive due to a lack of randomized controlled studies. This protocol describes a non-blinded, cluster-randomized controlled trial with the specific objective of testing the impact of reduced aflatoxin exposure on (individual) child linear growth.

**Methods/Design:**

Participants were recruited from among households containing women in the last 5 months of pregnancy in 28 maize-growing villages within Meru and Tharaka-Nithi Counties in Kenya. Households in villages assigned to the intervention group are offered rapid testing of their stored maize for the presence of aflatoxin each month; any maize found to contain more than 10 ppb aflatoxin is replaced with an equal amount of maize that contains less than this concentration of the toxin. They are also offered the opportunity to buy maize that has been tested and found to contain less than 10 ppb aflatoxin at local shops. Clusters (villages) were allocated to the intervention group (28 villages containing 687 participating households) or control group (28 villages containing 536 participating households) using a random number generator. The trial, which is funded by United Kingdom (UK) aid from the UK government, the Global Food Security Portal, and the Ministry for Foreign Affairs of Finland, is currently ongoing.

**Discussion:**

This study is the first randomized controlled trial (RCT) to test for a causal impact of aflatoxin exposure on child growth. Whether or not this relationship is found, its results will have implications for the prioritization of aflatoxin control efforts by governments in affected regions, as well as international donors.

**Trial registration:**

American Economic Association RCT Registry # 0000105. Initial registration date: 6 November 2013, last updated 30 December 2014.

**Electronic supplementary material:**

The online version of this article (doi:10.1186/s13063-015-1064-8) contains supplementary material, which is available to authorized users.

## Background

This protocol for the Mitigating Aflatoxin exposure to Improve Child growth in Eastern Kenya (MAICE) study, current as of 30 June 2015, is based on the SPIRIT (Standard Protocol Items: Recommendations for Interventional Trials) reporting guidelines as detailed by Chan et al. [1]. A completed SPIRIT Checklist for the study is included as Additional file 1. Aflatoxin is a naturally occurring toxic by-product, named after a genus of fungus that produces it (*Aspergillus flavus and Aspergillus parasiticus*). *A. flavus* colonizes food crops including maize; *A. parasiticus* is a concern in groundnuts as well as tree nuts (cashews and pistachios) and a range of other produce^1^. Adverse impacts of aflatoxin on human and livestock health are well-established. For example, acute exposure in humans can lead to liver disease, liver failure and death from aflatoxicosis [2]. Aflatoxin has been classified by the International Agency for Research on Cancer (IARC) as a Type 1 human carcinogen that causes hepatocellular carcinoma (liver cancer) [3]. Additionally, chronic exposure to aflatoxin among livestock reduces productivity and growth rates [4].

The impact of chronic aflatoxin exposure on child growth is not well-understood. In Ghana, women exhibiting high serum aflatoxin levels – a marker of having been exposed over the previous 2 to 3 months – at delivery were more likely to have a low-birth weight baby; no association was found with having a baby small for gestational age or with preterm birth [5]. The analysis controlled for socio-economic status (SES), but no details were provided on how this was done, making it difficult to evaluate whether confounding was adequately controlled for. A study in the Gambia showed that exposure occurred before the linear growth retardation: serum aflatoxin levels in pregnant women and in infants at 16 weeks of age were strong predictors of linear growth during the first year of life. Cord blood levels were not associated with birth weight or length [6]. The Gambia study did not control for SES. A study in Benin and Togo found that the serum aflatoxin level was 30–40 % higher in stunted children 1 to 5 years of age than in non-stunted children, after controlling for confounders including SES, child age, and sex. Details on the measure of SES were not provided by the authors [7]. Finally, the same authors studied the linear growth of 200 Togolese children 16 to 37 months of age over an 8-month period. Children in the highest serum aflatoxin albumin quartile grew 1.7 cm less than children in the lowest quartile, after controlling for age, sex, baseline length, and SES. As in the previous study, the authors did not provide details on the SES measure used [8].

Although these findings are generally consistent, none of the studies used a controlled intervention methodology. As a consequence, it is not clear to what extent the association between aflatoxin and child linear growth was confounded by factors such as household SES, child morbidity and dietary intake. For example, it may be the case that poor households are less able to afford proper post-harvest technologies to ensure that maize is fully dried and properly stored; this would potentially lead to poorer households consuming own-produced maize with higher levels of aflatoxin contamination. Poorer households may also consume more aflatoxin as a result of purchasing lower-quality maize. Poorer households also have lower dietary intake and diversity, poorer sanitation, and higher morbidity, which may manifest in stunted children, thereby inducing a spurious correlation between aflatoxin and child linear growth.

The specific objective of this study is to determine whether consumption of aflatoxin has a direct causal impact on child linear growth during the period from before birth to age 24 months. The hypothesized pathway is a biological one: human and animal studies indicate that aflatoxin causes immunosuppression (which in turn can lead to repeated infections and, consequently, growth retardation in young children), impairs protein synthesis, and causes changes in the hepatic metabolism of micronutrients [4]. It has also been suggested that aflatoxin, together with fumonisin and deoxynivalenol (two other mycotoxins commonly found in maize and groundnuts), mediate intestinal damage similar to environmental enteropathy [9]. This condition is known to lead to chronic systemic immune activation and malabsorption of nutrients, which in turn may lead to growth retardation.

Testing the hypothesis that aflatoxin exposure impairs child linear growth requires random assignment to reduced consumption of aflatoxin, keeping constant income, food availability, and other factors that affect child linear growth. A cluster-randomized design is necessary due to the risk that under an individual-level randomization, households assigned to the control group could potentially access tested, aflatoxin-safe maize, provided to those in the control group through the study. Despite the observed association between aflatoxin exposure and adverse health outcomes, enforcement of regulations to limit exposure is weak in many less developed countries. An intervention to systematically remove contaminated food from people’s diets as implemented for this study is not currently feasible outside of a research context due to logistical complexity and cost. Comparison of the intervention against status quo aflatoxin exposure is, therefore, justified.

## Methods

### Trial design

The study is based on a parallel-group design, with a 50:50 allocation of villages to the intervention and control groups by simple random assignment using a computer-generated sequence, implemented by the investigators. Villages (the unit of randomization) are defined according to their formal administrative boundaries.

### Study site and selection of clusters

The study site is rural Meru and Tharaka-Nithi Counties in Kenya, a known global hotspot for aflatoxin exposure through maize. Outbreaks of acute aflatoxicosis were reported in this region in 1981, 2004, 2005, and 2006, with widespread contamination of maize reported in 2010 [10–15]. Villages in these two counties, which were identified by a local agricultural expert as growing maize as the predominant crop, were eligible for inclusion in the study. Selection of these villages proceeded as follows: one village was randomly selected using Stata’s random number generator (StataCorp, College Station, TX, USA) for inclusion in the study. A second village was then randomly selected. Using the latitude and longitude of the villages, the second village was excluded from the study if it was within 4 km from the original village, otherwise it was retained in the study. This process continued, randomly selecting villages and retaining them only if they were not within 4 km of *any* other retained village, until 56 villages were selected for inclusion in the study. Due to the potential negative impact on maize farmers’ livelihoods of making aflatoxin exposure levels in certain villages public, the list of study villages is not publicly available.

### Sample size

To determine sample size, the minimum detectable effect (MDE) for exposure to treatment for the full 24-month period was set to 0.3 length-for-age Z-score (LAZ), based on the magnitude of effect sizes that have been obtained with known effective nutrition interventions [16]. This was then adjusted for partial exposure to the intervention of participants recruited in the first year of the study (prior to introduction of the stockist component) to give an MDE of 0.281.

Sample size calculations were performed to achieve an alpha of 0.05 and power of 80 % to detect a difference of means in LAZ between children in intervention and control villages using a 1-sided test. Based on anthropometric data collected in similar conditions, the standard deviation of the LAZ of children was assumed to be 1.28. Assuming an intra-cluster correlation for LAZ of 0.05, attrition of 9 %, and that 15 % of variation in the outcome would be explained by baseline variables, the estimated total number of participants required to achieve the study objectives was 924 across 56 villages

### Eligibility and recruitment of participants

Enrollment of participants within these villages was conducted by the African Population and Health Research Center in 2013 and by Innovations for Poverty Action in 2014. Within each of the study villages, enrollment into the study was conducted in 6 waves, each 4 months apart. In each wave, women in the fifth to final month of pregnancy (by the woman’s estimate) were recruited to participate in the study. Scouts employed by the data collection firm identified pregnant women in the study area before the survey team arrived. This was done 6 times over the course of recruitment, at 4-monthly intervals. Informed consent was obtained from all participants as follows. The consent form, included as Additional file 2, was read to each potential participant by an enumerator at the initial visit. The participant was then asked to sign the document if he or she agreed to participate. All pregnant women who gave informed consent in this way were enrolled in the study. The number of recruitment waves required to attain an adequate sample size was calculated using birth rates from 2008 to 2009 Kenya Demographic and Health Survey and populations of the study villages from the 2009 Kenya Census.

### Intervention

The intervention consists of two components: swap and stock. In the swapping component, households are visited monthly by trained staff of Caritas Meru, a non-governmental organization that works with farmers in the study area, and offered rapid aflatoxin testing of any stored maize that the household plans to consume over the next 2 months. If the household agrees, a composite sample of at least 150 grams of flour or 300 grams of whole kernels is taken from multiple regions of the container in which the identified maize is stored, and ground using a manual grinder, so that at least 70 % of the sample passes through a 20-mesh sieve, in accordance with US Grain Inspection, Packers and Stockyards Administration (GIPSA) aflatoxin testing protocols. A rapid aflatoxin test is then conducted using a 10-gram sub-sample of the homogenized composite sample using the GIPSA-verified Romer (Romer Labs®, Inc., USA) AgraStrip rapid test with a 10 parts per billion (ppb) detection threshold, according to manufacturer instructions. Any maize found to contain over 10 ppb aflatoxin (the Kenyan regulatory limit for aflatoxin contamination) is replaced with an equal amount of maize that has been tested and found to contain less than 10 ppb aflatoxin (“safe maize”). This component of the intervention has been in place since July 2013.

The stockist component of the intervention was introduced after trial commencement, in January–February 2014, in response to the observation that many households were accessing the majority of the maize they consumed through the market. This was unexpected and arose due to an unusually poor maize harvest in the study area in 2013. Household maize purchases are typically small and frequent, so the swapping intervention was missing a large portion of maize consumed. In the stockist intervention, Caritas Meru supplies maize containing less than 10 ppb aflatoxin to at least one shopkeeper in each of the intervention villages. In geographically larger villages, safe maize is supplied to multiple shopkeepers to ensure this maize is accessible to all study participants. Participating households are encouraged to purchase this tested maize, which is offered at the lowest price of maize currently for sale in the village. This encouragement is through an initial village meeting, as well as the monthly swapping visits. Participants have been provided with a laminated ID card displaying their name and unique identifying number. To ensure that an adequate supply of safe maize is available to study participants, stockists are asked to sell maize only to those presenting a study ID card, and to record all sales in a tracking form which includes a field for the household unique identifying number.

Participants in villages assigned to the intervention group were consuming untested, purchased maize for up to 12 months before the stockist component of the intervention was introduced, half of the study duration of 24 months. Due to the risk that these participants were exposed to the full intervention for an insufficient length of time to have an impact on the primary outcomes of interest, an additional wave of recruitment was added to replace this wave of recruits. Wave-1 households in the intervention group continued to receive the intervention for a 2-year period, according to the original protocol, but this group will be excluded from follow-up data collection.

### Outcomes and data collection

The primary outcomes are serum aflatoxin B1-lysine adduct level determined using HPLC analysis and linear growth (assessed using LAZ) of children aged 20–24 months. We focus on the period from before birth to age 24 months because growth faltering occurs primarily during this period [17], and the greatest benefits of nutritional interventions are seen at the youngest ages [18–21]. Data on household SES, food consumption, and child-feeding practices are also collected in order to control for these known determinants of child linear growth and hence reduce residual noise and increase power

Data collection at baseline and follow-up occurs at participants’ homes through face-to-face interviews. A pre-coded survey was administered to the expectant mother immediately after enrollment, her height and weight were measured, and self-reported month of pregnancy was recorded. Expectant mothers were also asked to provide a venous blood sample to be analyzed for serum aflatoxin. Additional file 3 provides details on the protocol for blood data collection and analysis of serum aflatoxin. A similar survey is be repeated during follow-up data collection at 24 months after enrollment. Participants enrolled in the fourth through sixth waves will additionally be followed-up 24 months after the third enrollment wave. At each follow-up visit, the length and weight of the child in utero at baseline (reference child) will be recorded, and a venous blood sample will be taken from this child for serum aflatoxin analysis.

**Fig. 1:**
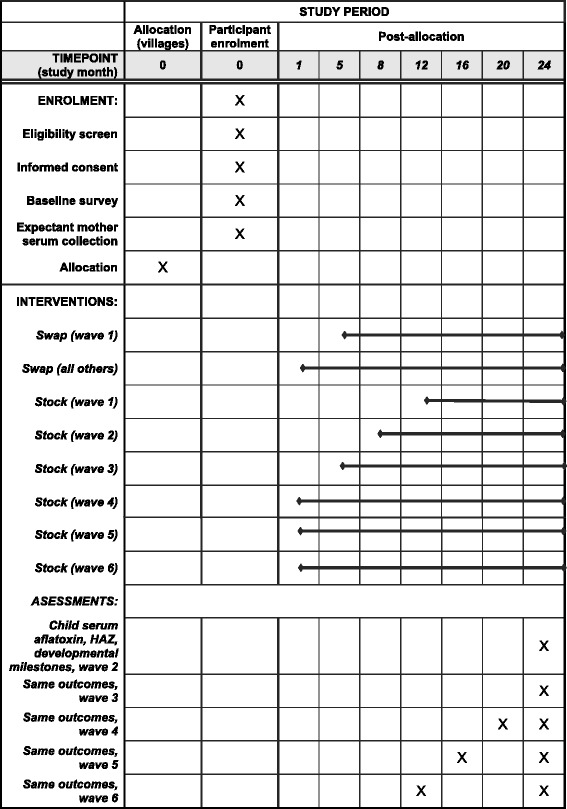
Schedule of enrollment, interventions, and assessments. X: discrete point in time during which an event occurs. : continuous period over which an event occurs

Participants will be tracked for follow-up data collection using global positioning system (GPS) data on the homestead location and respondent’s phone number collected at enrollment. If the target child has relocated since enrollment, the child will be followed to the new home if this is within a sub-location (administrative unit above the village) included in the study. If the target child has relocated outside of the study area, or is no longer living, but the household can be tracked and contains another child aged between 12 and 24 months, primary outcome data will be collected for this child. Data will be collected for all participants tracked during follow-up data collection, regardless of whether any of their stored maize was swapped or whether they purchased tested maize from the stockist (Fig. 1).

Data from baseline and follow-up surveys is collected using handheld tablets on which an electronic data collection form is programmed. The program includes range and consistency checks, and automatic skip patterns. Backcheck surveys consisting of a subset of the survey questions are administered to randomly selected participants to ensure data quality. Tablets are sent to the field office on a regular basis where the data is removed for secure storage.

### Study timeline

The first wave of enrollment into the study began in February 2013, and enrollment of the final wave (wave 6) was completed in November 2014. Follow-up data collection on wave-2 enrollees began in July 2015. Data collection for all waves is expected to be completed by November 2016.

### Data analysis plan

Data will be analyzed on an intent-to-treat basis, meaning that non-compliers (participants who refused to allow their stored maize to be tested and swapped, or who did not purchase any aflatoxin-safe maize from stockists) are treated as part of the intervention group for purposes of analysis. Both unadjusted and adjusted analysis of covariance (ANCOVA) models will be used to assess the impact of the intervention, controlling for mothers’ blood aflatoxin level at enrollment. In all models, standard errors will be clustered at the village level to account for within-cluster correlation of outcomes.

### Ethics and dissemination

Ethical approval was obtained from the International Food Policy Research Institute (IFPRI) Institutional Review Board, and from the African Medical Research Foundation Ethics and Scientific Review Committee in Kenya, and has been updated with changes in the protocol after commencement. Approval of changes to protocol modifications are submitted to the same. Adverse effects, while not anticipated, would be reported to both of these bodies immediately.

Identifiers will be used to uniquely identify individuals and households. Once the data sets have been created, any information allowing the identification of an individual or household (such as names and address) will be stripped from the data sets and destroyed. Any information allowing the identification of individuals or households will be destroyed. Any information obtained in connection with this study will be used in a manner that does not publicly disclose any participant’s identity and will be kept confidential. Only study investigators will have access to the final data set.

Serum aflatoxin levels at enrollment are reported back to participants during village-level dissemination meetings and household visits several months later. Summary statistics of the baseline blood and maize aflatoxin results have been reported to the Kenyan Ministry of Agriculture, Ministry of Health, and to the Meru and Tharaka-Nithi County administration offices. Full results will be provided to the same government bodies, and submitted for publication in peer-reviewed journals. Eligibility for authorship will be based on substantive contribution in three of five of the following areas: inception, fundraising, data collection, analysis, and manuscript preparation.

## Discussion

This protocol describes the first randomized controlled trial (RCT) to test for a causal impact of aflatoxin exposure on child growth. Given the strength of this relationship in published observational studies, the hypothesized health burden associated with an impact on growth, if present, would almost certainly exceed the known burden due to liver cancer and acute toxicity. Whether or not such relationship is found, results of the trial will have implications for the prioritization of aflatoxin control efforts by governments in affected regions, as well as international donors, which have been investing heavily in aflatoxin control in recent years [22].

The strength of this study compared to previous work addressing the same question is its utilization of a randomized controlled design. This approach overcomes the problem of omitted variable bias: that is, the possibility that the observed relationship between aflatoxin exposure and impaired child growth is driven by confounders. In order to reduce aflatoxin exposure among those assigned to the control group, the intervention provides participants with opportunities to have their stored maize tested and replaced with aflatoxin-safe maize if contaminated, and to purchase aflatoxin-safe maize through local stockists. The primary risk to the study is a lack of demand for these services: if participants in the treatment group cannot be reached, or do not submit to having their stored maize tested and swapped each month, or if they purchase maize from non-study vendors, aflatoxin exposure may not be sufficiently reduced to impact child growth outcomes. A further risk is lack of information on serum aflatoxin throughout the 2-year study period. Due to budget constraints, serum aflatoxin levels are only collected at enrollment (from expectant mothers) and from children at one or two follow-up visits, depending on enrollment wave. The difference in aflatoxin exposure achieved through the intervention over the study period as a whole will be estimated using data on the proportion of stored maize swapped and the proportion of purchased maize obtained through study-affiliated stockists. Future trials using a similar design would ideally collect serum samples from all children at multiple points in time over the study period to more precisely estimate exposure.

## Trial status

At the time of submission, recruitment had been completed and the intervention was ongoing. Follow-up data collection had not yet begun.

## Additional files

Additional file 1:
**SPIRIT 2013 Checklist.** A list of recommended items to address in a clinical trial protocol and related documents, based on the 2013 SPIRIT (Standard Protocol Items: Recommendations for Interventional Trials) guidelines. (DOC 120 kb) Additional file 2:
**Consent form.** A description of study aims, procedures, risks, and benefits read to prospective participants during the informed consent process. (DOCX 65 kb) Additional file 3:
**Protocol for blood data collection and analysis of serum aflatoxin.** Protocol for blood data collection from study subjects and subsequent analysis of serum for aflatoxin. (DOCX 18 kb)

## Abbreviations

ANCOVA: analysis of covariance
GIPSA: Grain Inspection, Packers and Stockyards Administration
GPS: global positioning system
IARC: International Agency for Research on Cancer
IFPRI: International Food Policy Research Institute
HPLC: high-performance liquid chromatography
LAZ: length-for-age Z-score
MAICE: Mitigating Aflatoxin exposure to Improve Child growth in Eastern Kenya
MDE: minimum detectable effect
ppb: parts per billion
RCT: randomized controlled trial
SES: socio-economic status
SPIRIT: Standard Protocol Items: Recommendations for Interventional Trials
UK: United Kingdom

## Footnotes

1 Other affected produce include sorghum, figs and spices; meat, milk and eggs may contain lower levels of aflatoxin and its metabolites.
