# Supplementary material for: Mitigating aflatoxin exposure to improve child growth in Eastern Kenya: study protocol for a randomized controlled trial
Source: Trials. 2015 Dec 3;16:552. doi: 10.1186/s13063-015-1064-8 (PMC4669614; doi:10.1186/s13063-015-1064-8)
Supplement: Additional file 2: — Consent form. A description of study aims, procedures, risks, and benefits read to prospective participants during the informed consent process. (DOCX 65 kb) [file 13063_2015_1064_MOESM2_ESM.docx]

**Informed Consent Form**

| **Study Title** | Mitigating aflatoxin exposure to improve child growth in Eastern Kenya |
| --- | --- |
| **Investigator(s)** | Vivian Hoffmann, International Food Policy Institute, Washington, DC, USA. +1 202 862 8169  Kelly Jones, International Food Policy Institute, Washington, DC, USA. +1 202 862 4641 |
| **Study Sponsor(s)** | MTT; DFID |
| **Collaborators** | Innovations for Poverty Action – Kenya  International Livestock Research Institute  University of Nairobi |

**This Informed Consent Form has two parts:**

• **Information Sheet (to share information about the study with you)**

• **Certificate of Consent (for signatures if you choose to participate)**

**You will be given a copy of the full Informed Consent Form**

**Part I: Information Sheet**

The International Food Policy Research Institute is conducting research on the health effects of aflatoxin in maize, and storage practices that may help reduce levels of aflatoxin contamination. We are giving you this information because we would like you to participate in our research project. If you prefer not to participate, you are free to choose to do so. There is no negative impact of not participating. We want to make sure that you have all the information that you need before you decide. Members of our team are here to help you understand more about the project. If you do not understand any of the words or ideas that you see on this form, please ask us to explain the information to you. You can talk to anyone from our team whom you feel comfortable with about the research.

**Why is this Project Important?**

Aflatoxin is known to be fatal in large quantities and is a known carcinogen, but the broader health implications of long-run exposure to moderate quantities are not well understood. Some people have suggested that when children under two years of age consume aflatoxin it limits their growth. Showing whether this is the case is important in making sure that everyone (individuals, the government, NGOs) works hard to tackle the problem of aflatoxin. This study will help us understand the wider health implication of aflatoxin.

We also need to know how tackle the problem of aflatoxin. This study will test new storage technologies to see if they can help reduce aflatoxin levels.

**Who Can Participate?**

You are being invited to take part in this research project because you are about to be a mother of a young child or because you have a child in your household under the age of 2 years.

**Participation is Your Choice**

Your participation in this research is completely voluntary. You will make the choice about whether you will participate or not. If you choose not to take part, you will continue to receive all of the services that you usually get in your community and nothing will change.

**What Is Involved in this Project?**

If you agree to participate in the project, the following will happen:

1. In the next week we will ask you to complete a survey about your family, your crops, your health and the health of your children. The interview may last for over two hours. If you have a child under age 2, we will also measure his/her height and weight. In addition, we would like to take 2ml of blood from you. This will be done quickly, will not cause any harm and will cause only minor discomfort to you. The blood will be used to test your aflatoxin levels. Because of the lack of adequate lab in Kenya to carry out the type of test we are interested in, the sample blood taken from you will be sent to the US for analysis. After blood tests are done any remaining blood sample will be destroyed. You will be informed of the results. Your participation in the interview is voluntary. You have the right to not participate if you don’t want to. You also have the right to refuse to answer specific questions or to discontinue the interview at any time. Not participating will not affect you, your children or your family.
2. You will be invited to attend a training session at which information on the health effects is provided and some training is given on harvest and post-harvest practices that can reduce aflatoxin in maize.
3. You will be assigned to group A, B or C. All households in your village that are participating in this study will be assigned to the same group. We have assigned your village to one of these groups by random selection.
4. If you are in group A you will participate in further training on harvest and post-harvest practices and be offered a post-harvest technology. In this case we will test your maize four months after harvest.
5. If you are in group B you will have your maize tested every two months for two years and swapped for aflatoxin free maize if it is found to have high levels of aflatoxin.
6. If you are in group C you will have your maize tested after harvest.
7. In 24 months’ time we will ask you to complete another survey about your family, your crops, your health and the health of your children. The interview may last for over two hours. At the same time we will measure the height and weight of any children born to you between now and then. In addition, we might ask to take a small amount of blood from your young child. This will be done quickly, will not cause any harm and will cause only minor discomfort to the child. The blood will be used to test the aflatoxin levels of your child. After blood tests are done any remaining blood sample will be destroyed. The results of the test will be shared with you. Your participation in the interview and blood draw is voluntary. You have the right to not participate if you don’t want to. You also have the right to refuse to answer specific questions or to discontinue the interview at any time. Not participating will not affect you, your children or your family.
8. We will hold a meeting to explain the results of the study.

If changes are made to the study or new information becomes available, you will be informed.

**How Long will the Project Last?**

This study takes place over 3 years.

**What are the Risks?**

There is a risk that you may share some personal or confidential information by chance, or that you may feel uncomfortable talking about some of the topics in this study. However, we do not wish for this to happen. You do not have to answer any question or take part in the survey if you feel the question(s) are too personal or if talking about them makes you uncomfortable.

**What are the Benefits?**

You will be given information about your child’s anthropometric status at 24 months and aflatoxin exposure of you and your child. In addition to this direct benefit to you, your participation is likely to help us find out more about the health effects of aflatoxin and how to reduce aflatoxin levels by improved storage technologies. This will help us encourage people to address the problem of aflatoxin.

**How will we Protect your Information and Confidentiality?**

The research being done in the community may draw attention and if you participate you may be asked questions by other people in the community. We will not be sharing information about you or your children to anyone outside of the research team. The information that we collect from this research project will be kept private. Any information about you will have a number on it instead of your name. Only the researchers will know what your number is and we will lock that information up with a lock and key. It will not be shared with or given to anyone outside of our project.

**What will Happen with the Results**

The knowledge that we get from this research will be shared with you and your community before it is made widely available to the public. Each participant will receive a summary of the results. There will also be small meetings in the community and these will be announced. Following the meetings, we will publish the results so that other interested people may learn from the research.

**Can I Refuse to Participate or Withdraw from the Study?**

You do not have to take part in this research if you do not wish to do so. If you choose not to participate, you will continue to receive all of the normal services that you usually get and nothing will change. If you wish to stop participating in the study after you begin, you can stop at any time by telling someone on our project team. If you choose to stop taking part, you will continue to get all of the normal services that you usually get in your community.

**Who Can I Contact?**

If you have any questions, you can ask anyone from our team now or later. If you have questions later, you may contact Vivian Hoffmann, v.hoffmann@cgiar.org, Tel: +1 202 862 8169 or Lulu Tian, [ltian@poverty-action.org](mailto:ltian@poverty-action.org), +254 700 726 544.

If you have questions about your rights as a research participant, you may contact:

The Research Officer, AMREF Kenya

Wilson Airport, Lang’ata Road

P.O Box 30125-00100, Nairobi, Kenya

Office Tel: +254 20 6994000; Fax: +254 20 606340

**Do you have any questions at this time?**

**Part II: Certificate of Consent**

**I have read the above information, or it has been read to me. I have had the opportunity to ask questions about it and any questions I have been asked have been answered to my satisfaction. I consent voluntarily to be a participant in this study.**

| **Print Name of Participant** | [at least forename and surname] |
| --- | --- |
| **Signature of Participant** |  |
| **DD/MM/YYYY** |  |

[A literate witness must sign and should be selected by the participant and MUST have no connection to the research team.]

**I have witnessed the accurate reading of the consent form to the potential participant, and the individual has had the opportunity to ask questions. I confirm that the individual has given consent freely.**

| **Print Name of Witness** | [at least forename and surname] |
| --- | --- |
| **Signature of Witness** |  |
| **DD/MM/YYYY** |  |

**Statement by the researcher/person taking consent**

**I have accurately read out the information sheet to the potential participant, and to the best of my ability made sure that the participant understands the steps in the research project outlined above. I confirm that the participant was given an opportunity to ask questions about the study, and all the questions asked by the participant have been answered correctly and to the best of my ability. I confirm that the individual has not been coerced into giving consent, and the consent has been given freely and voluntarily.**

**A copy of this ICF has been provided to the participant.**

| **Print Name of Researcher/person taking the consent** | [at least forename and surname] |
| --- | --- |
| **Signature of Researcher/person taking the consent** |  |
| **DD/MM/YYYY** |  |
