# Supplementary material for: Mitigating aflatoxin exposure to improve child growth in Eastern Kenya: study protocol for a randomized controlled trial
Source: Trials. 2015 Dec 3;16:552. doi: 10.1186/s13063-015-1064-8 (PMC4669614; doi:10.1186/s13063-015-1064-8)
Supplement: Additional file 3: — Protocol for blood data collection and analysis of serum aflatoxin. Protocol for blood data collection from study subjects and subsequent analysis of serum for aflatoxin. (DOCX 18 kb) [file 13063_2015_1064_MOESM3_ESM.docx]

**Protocol for blood data collection and analysis of serum aflatoxin**

Protocol for sample collection in the field:

- - - 1. Collect 1.5 to 2 ml of blood using a 22 gauge syringe in serum tubes with clotting gel. Make sure the tubes are adequately labelled.
      2. Invert carefully 5-6 times to mix clot activator.
      3. Store tubes in an ice chest (which is filled with fresh ice each morning).
      4. At night, centrifuge for 15 min at manufacturer’s recommended speed (usually 1000-2000 RCF). Do not use brake to stop centrifuge.
      5. Carefully aspirate the supernatant (serum) at room temperature and pool into a centrifuge tube using a disposable Pasteur pipette. Take care not to disturb the cell layer or transfer any cells. Use a clean pipette for each tube.
      6. Inspect serum for turbidity. Turbid samples should be centrifuged and aspirated again to remove remaining insoluble matter.
      7. Aliquot into 2 cryovials and store in chest with dry ice. Ensure that the cryovials are adequately labelled. (CRYOVIAL: Nunc Linear Barcoded (inkjet,code 128) CryoTube Vial, 1.8 mL, int thread, round bottom, self-standing, anti-rotation, PP tube ([www.thermoscientific.com)](http://www.thermoscientific.com)))
      8. Transfer samples to Nairobi on dry ice.
      9. Store samples in freezer on ILRI campus and ship to the US on dry ice.

Protocol for analysis of the serum samples:

- - - 1. Serum aflatoxin B1-lysine adduct level will be determined using HPLC analysis.
      2. Analysis will be conducted at the University of Georgia. Analysis will use current best practice lab procedures ensuring the highest possible precision and accuracy. This will include (but not be limited to) the addition of spiked samples for every 5 samples processed and addition of normal human serum without aflatoxin adducts.
